# A Position They Soon Must Hold

**Published:** 1920-11-27

**Authors:** 


### THE ISOLATION HOSPITALS.

### A Position They Soon Must Hold.

There is an interesting side to the problems of hospital reorganisation which was ably put forward at a recent meeting of the Royal Society of Medicine by Dr. A. K. Chalmers, the medical officer of health of Glasgow. In dealing with the past history of isolation hospitals and the exact proofs of value which they have already given, it was obviously necessary that the speaker should collect and bring forward a number of statistical records. It is impossible, however, in this short note to consider these in any detail, and it must suffice to take for granted, in a general sense, the progressive reduction in infectious disease arising with fuller notification and corresponding isolation of the cases.

In sketching this gradual development Dr. Chalmers considered chiefly the fluctuations of prevalence corresponding to *degree* of isolation reached at any particular period, and, as he rightly explained, if this relationship were simple, then a high percentage of isolation would mathematically correspond to a low prevalence, and the approach could be prophesied of a period when the volume of infectious disease would be practically negligible. *Vice versa*, a careless isolation service would produce dire results in the not distant future. But in practice there have been many minor fluctuations of prevalence which ran distinctly counter to both these deductions, and it is upon such small sections that many partial opponents of the strict isolation principle have based their arguments.

Obviously, we must in justice consider the whole period covered, and it is then seen that the position of the isolation hospital and value of an increasing degree of infectious disease isolation are fully established. On the other hand, it must be realised that periodically disease appears to leap the barriers of isolation, and then for a time we are beset with problems and features in the natural history of the disease with which the fever-hospital principle as such certainly fails completely to cope.

Dr. Chalmers, citing the incidence of diphtheria, says: "In epidemic periods not only does its volume rise above the lower limits of scarlet fever, but there is an interesting correspondence between the epidemic prevalence of both in the period 1904-17 which recalls the eighteenth-century uncertainty as to the difference between these diseases. Its isolation rate was approaching 60 per cent. when the increased prevalence began, and had reached 80 per cent. in 1905, when the epidemic entered upon its most active phase. Isolation was then a well-established practice before the acute epidemic phase began; but again, as in scarlet fever, diphtheria unrolled another page of history and then receded, leaving us to interpret the writing as best we may."

And certain it is in this process of interpretation that the greatest advances in the study of infectious disease have arisen. Results have sometimes come from the by-ways of observation, but generally painstaking investigation of "return" cases, lines of spread, contacts, etc., have been the chief source. The essential facts concerning purely "carrier" cases have been realised, and they, as in the late war, are being gradually eliminated. The intimate part played by insect transmission is becoming more accurately understood, and most of the facts concerned are among the now accepted tenets of epidemiology. But still there remain a number of quite unexplained cross-infections and minor outbreaks within the wards of the isolation hospitals themselves.

But on the prevalence of disease among the public at large this last section has not so direct an influence, and its occurrence in no way detracts from the value of isolation as a protective measure to the community and a preventive measure as far as the disease is concerned. The somewhat anomalous situation which persists to-day is that nothing approaching a general policy concerning isolation is either accepted or practised. Some authorities move to hospital less than half their cases of scarlet fever. In some hospital towns, especially those having a teaching centre, typhoid fever is admitted to general hospitals; in other centres hospitals adopt a practice of rigidly excluding everything in the nature of infectious disease. Sometimes neither measles nor whooping-cough may be treated in hospitals. Elsewhere erysipelas when dealt with may be relegated to a Poor-law institution, puerperal fever to a hospital for women, ophthalmia and trachoma to an infirmary.

As illustrative of these variations, Dr. Chalmers makes a comparison of the annual cost of isolation hospitals in different areas. Taking four large cities, we find the estimated annual outlay on isolation for the present year to be respectively 3s. 5d., 3s. 1d., 1s. 11d., and 1s. 3d. per head of population. It is true that volume of disease affects these figures, but local custom is the more potent element, and although it must also be admitted that the social class dealt with and the cleanliness of the average home form further deciding factors in the official expenditure necessary, yet one feels that experience in the past suggests that there will, for a long time, be so high a percentage of unhygienic homes that greater special hospital provision is the requirement of the moment.

It appears to some extent that the cost has made custom include only the diseases which *must* be isolated. There rarely arises that situation where really adequate accommodation allows of wholesale testing and observation on a large scale. The policy has been to do without it if possible. Thus it is not surprising to read that "if a united front is to be presented to disease, all infections, and not only some, will require consideration, and

**The Isolation Hospitals**—(*continued*).

growth of knowledge may well bring some of the parasitic skin infections within the range of preventive action."

In the large number of illustrations which Dr. Chalmers gives of epidemic spread outstripping isolation, he claims emphatically that it is not isolation itself which has failed. Rather has the weakness been due to inability efficiently to supplement it by secondary prevention. Also, circumstance has in many instances separated the fever hospitals from the teaching schools and correspondingly restricted the investigational work which might be carried on in connection with them, pinning the institutions themselves down strictly to isolation duties as such and direct treatment of their cases. As this officer rightly claims, the increasing variety of diseases notifiable and coming within the fever-hospital sphere makes the development of clinical research therein an absolute necessity. "In the interest alike of the community, the State, and the training of future practitioners of medicine, it seems desirable to contemplate a time when the epidemic hospital will become an institute for research," and this view is deserving of full recognition in any national hospital schemes which are brought forward. Not only must the isolation hospital be included, but it must be given a proportionately higher importance and opportunity for development. Many attacks upon medical problems in the country call for a more adequate institutional background, but few more insistently than that of epidemic disease.

